# Bridging silos on a budget: how interprofessional education shapes collaborative attitudes across low-and middle-income countries - a systematic review

**DOI:** 10.1186/s12909-025-08203-6

**Published:** 2025-11-14

**Authors:** Urvish Joshi, Renjith Raj Puthuparampil, Sanjay Kini, Sharon Baisil

**Affiliations:** 1 Department of Community Medicine, Narendra Modi Medical College & LG Hospital, Ahmedabad, Gujarat India; 2 Saveetha Institute of Medical and Technical Sciences, Chennai, India; 3 Department of Community Medicine, Kasturba Medical College, Manipal, Manipal Academy of Higher Education, Manipal, , Karnataka India; 4 Department of Community Medicine, Malankara Orthodox Syrian Church Medical College, Kolenchery, Kerala India

**Keywords:** IPE, Collaborative attitudes, LMIC, Health-profession students, Systematic review, Narrative synthesis, PRISMA

## Abstract

**Background:**

Interprofessional education (IPE) enables health-profession students from different disciplines to learn together, fostering teamwork, communication, and collaborative practice. Although widely implemented in high-income countries (HICs), evidence from low-and middle-income countries (LMICs) is limited and scattered. This systematic review synthesized quantitative evidence on the impact of IPE interventions on students’ collaborative attitudes in LMIC contexts, and explored intervention characteristics, methodological quality, and evidence gaps.

**Methods:**

Following PRISMA (2020) guidance, we searched PubMed, Scopus, and Embase (January 2010-August 2025) and screened grey literature. Eligible studies included undergraduate or pre-licensure health-profession students from at least two disciplines in LMICs, exposed to structured IPE interventions, with pre- and post-measurement of attitudes using validated or described quantitative instruments. Two reviewers independently screened, extracted data, and assessed risk of bias using the Joanna Briggs Institute checklist for quasi-experimental studies. Certainty of evidence was rated with GRADE. Heterogeneity precluded meta-analysis; findings were synthesized narratively using the SWiM vote-count approach.

**Results:**

Five studies (Brazil, Iran, India, Lebanon, Pakistan; total baseline *N* = 396) met inclusion criteria. All used uncontrolled pre-post designs; three employed validated scales (IEPS, IPAS, RIPLS). Four studies reported statistically significant positive changes in overall attitudes: particularly in domains such as teamwork, role understanding, and communication. One reported a decline in a single patient-centeredness domain. Interventions ranged from single-day workshops to semester-long courses, delivered in classroom, blended, or clinical settings. Positive shifts were most common when IPE was embedded in authentic clinical environments and small-group learning. Risk of bias was moderate to serious, mainly due to lack of control groups, attrition, and reliance on self-report. Certainty of evidence was low.

**Conclusions:**

Structured IPE in LMIC undergraduate settings can improve collaborative attitudes, particularly when clinically embedded and professionally diverse. However, evidence is constrained by methodological limitations, small samples, and short follow-up. Future research should adopt controlled designs, harmonized outcome measures, and longer-term evaluation to assess sustainability and practice impact.

**Supplementary Information:**

The online version contains supplementary material available at 10.1186/s12909-025-08203-6.

## Introduction

IPE is recognized worldwide as an effective method for enhancing collaborative skills, patient safety, and health outcomes. It involves instances where students from multiple health professions learn about, from, and with each other to facilitate effective collaboration and improve health outcomes [1]. The World Health Organization’s (WHO) 2010 Framework for Action on IPE & Collaborative Practice advocates for the integration of IPE into health professional curricula to strengthen teamwork, communication, and ethical behavior [1]. HICs have made notable progress in adopting IPE, supported by established frameworks such as the Interprofessional Education Collaborative (IPEC) competencies from 2016 to 2023, which define essential areas like roles and responsibilities, interprofessional communication, teamwork, and values and ethics for collaborative practice [2, 3]. Conversely, evidence from LMICs is sparse and fragmented, with descriptive studies identifying barriers and facilitators but often lacking thorough outcome evaluation, especially for undergraduate and pre-licensure health profession students [4, 5]. 

Systematic reviews of IPE frequently emphasize attitudinal outcomes, such as willingness to collaborate, role comprehension, and communication skills, as immediate indicators of effectiveness, which are especially critical in LMICs due to resource constraints necessitating efficient team-based care models [1, 5]. However, most evidence derives from HICs using robust designs, while LMICs face scarcity, uncontrolled pre-post studies, lack of follow-up, and self-report biases, underscoring the need for focused synthesis on undergraduate/pre-licensure students where attitudes form early and influence future practice [5–7]. In HICs, IPE has shown sustained improvements in professional attitudes and collaborative behaviors among both students and practicing professionals, whereas graduate and continuing education often builds on existing silos; thus, this review targets undergraduates in LMICs to address the evidence gap and inform early curricular integration [6–8]. 

In a more recent systematic examination, Berger-Estilita et al. (2020) confirm that even in well-funded environments, assessments of medical students’ attitudes are hindered by ceiling effects and the inconsistent application of validated tools [6]. Similarly, Kitema et al.’s (2024) systematic review in sub-Saharan Africa points out issues such as limited outcome reporting, small sample sizes, and inconsistent study designs, underscoring the necessity for synthesizing quantitative changes in attitudes within LMIC contexts [5]. 

These results highlight a significant gap: there is no comprehensive synthesis quantifying IPE’s impact on attitudes in LMICs, the types of designs used, and methodological limitations that complicate interpretation. This systematic review synthesizes evidence on structured IPE interventions’ impact on collaborative attitudes among undergraduate health profession students in LMIC contexts, exploring intervention characteristics, methodological quality, and evidence gaps.

## Methodology

### Review Question, objectives and framework

Our primary research question aimed to explore how IPE interventions affect health-professional students’ attitudes towards collaborative practice in LMICs, and under what contextual conditions these effects occur. The main objective was to assess whether IPE led to a positive (↑), negative (↓), or neutral (↔) change in student attitudes, utilizing validated instruments to classify and code these outcomes. Secondary objectives included investigating how elements such as the context of delivery, the level of learners, and regional or cultural traits impacted these attitude shifts through narrative comparison; integrating qualitative themes to corroborate observed variations; assessing the risk of bias at the study level; summarizing the overall certainty of the evidence; and identifying evidence gaps for future research.

This systematic review used the PICO-framework to ensure a methodical approach to formulating questions, conducting literature searches, and selecting studies. The review explored: For undergraduate or pre-licensure health-profession students in LMICs, how do structured IPE interventions, compared to other interventions or none, influence attitudes toward interprofessional collaboration? The population consisted of undergraduate or pre-licensure students from at least two health professions, with studies taking place in LMICs as classified by World Bank FY-2026 income thresholds [9]. The intervention was a structured IPE activity, aligned with WHO (2010) definition and IPEC core competencies, where students learn with, from, and about each other in academic or clinical environments [1]. Comparators could be any educational activity or none, as seen in within-group pre/post designs. The outcome was quantitative change in attitudes toward interprofessional collaboration, assessed using validated instruments such as the Interdisciplinary Education Perception Scale (IEPS), Interprofessional Attitudes Scale (IPAS), Readiness for Interprofessional Learning Scale (RIPLS), Knowledge-Attitude-Practice (KAP) questionnaire, or a custom tool reporting numerical pre/post change [10–13]. 

The PICO-framework was enhanced by insights from the WHO Framework for Action on IPE & Collaborative Practice (2010) and the IPEC-Core Competencies for IPEC Practice (2016 update; 2023 revision), which highlight the importance of values and ethics, roles and responsibilities, interprofessional communication, and teamwork [1–3]. These global standards guided both the selection criteria and the analysis of the evidence base.

### Eligibility criteria

Studies were eligible if they met these conditions: The design had to be experimental, quasi-experimental, or interrupted time-series, with pre- and post-intervention measurements of student attitudes toward interprofessional collaboration. The population comprised undergraduate or pre-licensure students in health-profession programs like medicine, nursing, pharmacy, physiotherapy, or public health, representing at least two disciplines. Studies had to be conducted in LMICs as defined by World Bank FY-2026 income thresholds [9]. The intervention was a structured IPE activity such as workshops, courses, or simulations involving interactive learning across professions; facilitating learning with, from, and about multiple health professions, delivered in academic or clinical environments. The primary outcome was quantitative change in attitudes toward interprofessional collaboration, measured using a named, described, or validated scale-such as IEPS, IPAS, RIPLS, or KAP - or a custom tool reporting numerical pre/post change [10–13]. Only studies published in English between January 2010, and July 2025 were eligible.

Research was not included if it consisted of cross-sectional surveys, single-arm opinion polls, or was entirely qualitative. Studies that concentrated exclusively on postgraduate or licensed health professionals were deemed ineligible, as were those that did not conduct both pre- and post-intervention evaluations of attitudes. Additionally, non-primary research outputs such as conference abstracts, editorials, commentaries, and opinion pieces were excluded. Studies were also not considered if they did not meet the requirement of being set in LMICs or if they did not include participants from at least two different health professions.

### Design and registration

This systematic review adhered to the PRISMA (2020) guidelines for systematic reviews and meta-analyses [14]. All the items as per PRISMA checklist are mentioned in Appendix. The review protocol was planned in advance and registered with the Open Science Framework (OSF) under the registration number 10.17605/OSF.IO/63ZXD. A mixed-methods convergent integrated synthesis was utilized in the review to merge quantitative and qualitative evidence regarding IPE interventions in LMICs.

### Information sources and search strategy

Databases were searched from January 2010 to August 10, 2025, including PubMed/MEDLINE, Scopus, and EMBASE. A combination of controlled vocabulary (e.g., MeSH) and free-text terms related to “IPE,” “collaborative attitudes,” and “low- and middle-income countries” was used. The full database-specific search strategies are detailed in the Appendix 1.

To supplement the database search, we explored grey literature sources such as Google Scholar, OpenGrey, and institutional repositories. We also hand-searched the reference lists of all included studies and relevant systematic reviews to identify any additional eligible articles.

### Study selection process

All retrieved citations were imported into Rayyan for deduplication and screening [15]. Three reviewers (UJ, RRP, SB) independently screened all titles and abstracts for eligibility. Full texts for potentially relevant studies were then retrieved and independently assessed by both reviewers. Discrepancies were resolved through discussion, with a fourth reviewer (SK) arbitrating when necessary. The study selection process is summarized in a PRISMA flow diagram under the results section.

### Data extraction

Data were extracted independently by two reviewers (UJ, RRP) using a piloted extraction form. The extracted items included study identifiers, country and World Bank income classification, participant demographics, professional mix, and prior exposure to IPE. We also collected detailed information on intervention characteristics, such as the type, framework used, duration, delivery mode, setting, group size, professions included, facilitator details, and clinical focus. Outcome-instrument properties, numeric results, and any qualitative feedback were also extracted.

Discrepancies between reviewers were resolved through discussion or, when necessary, with the arbitration of a third or fourth reviewer (SB or SK). Where articles lacked certain information (e.g., attrition rates or reliability coefficients), this was coded as “NR” (Not Reported) to ensure transparency, in line with best-practice guidelines.

We extracted the following variables: author(s), year, country, income classification, participant disciplines, sample size, study design, intervention description (duration, setting, pedagogy), outcome instrument(s), timing of measurement, main results, and funding source.

### Risk-of-bias & certainty of evidence - assessment

The risk of bias for all included studies was assessed independently by two reviewers using the JBI Critical Appraisal Checklist for Quasi-Experimental Studies [16]. This tool evaluates potential bias across nine domains, including confounding, participant selection, classification of interventions, deviations from intended interventions, missing data, and reliability of outcome measurement. Disagreements between reviewers were resolved through discussion or third-party adjudication. The judgments for each study were presented item-by-item in a summary table and visually summarized using a colour-map.

Certainty of evidence for each outcome was assessed using the Grading of Recommendations, Assessment, Development, and Evaluation (GRADE) approach, considering risk of bias, inconsistency, indirectness, imprecision, and publication bias. Summary of findings tables were generated accordingly [17]. 

### Effect measures and data synthesis

Meta-analysis was not feasible due to heterogeneity in study designs, outcome measures, and reporting. Instead, we performed a narrative synthesis following the Synthesis Without Meta-analysis (SWiM) guideline [18]. Our approach involved grouping studies by learner level and delivery context, and coding primary attitude outcomes by effect direction (↑ for significant improvement, ↓ for significant decline, and ↔ for no change) using a p-value threshold of < 0.05. We visualized these vote-count results in a harvest plot, where the bar height was proportional to the paired-sample size. The narrative synthesis explored how delivery format, clinical exposure, and professional mix influenced the direction of effects. We also mapped evidence gaps by plotting intervention type against learner level. The findings were further grouped by geographic region, intervention type, and the specific instrument used, and we tabulated study characteristics and main outcomes, integrating qualitative descriptions where available.

### Publication bias assessment

We did not formally assess publication bias due to the small number of included studies (< 10) and heterogeneity in outcomes; however, we acknowledge the possibility of selective reporting in the included literature.

### Qualitative integration

Where available, open-ended responses were extracted and analyzed thematically. We used a convergent integrated mixed-methods approach, in which qualitative themes were triangulated with quantitative effect direction to identify plausible explanations for heterogeneity.

### Patient and public involvement

No patients or members of the public were involved in the design, conduct, reporting, or dissemination of this review.

## Results

### Study selection

Our searches identified 1,904 records (PubMed = 437, Scopus = 1,138, Embase = 329). After removing 384 duplicates, 1,520 unique titles/abstracts were screened; 1,458 were excluded at this stage for reasons such as: clearly not IPE, single-discipline/uni-professional reports, wrong publication type (e.g., editorials, protocols, commentaries), or not being an empirical intervention study.

Sixty-two full texts were retrieved and assessed for eligibility; 57 were excluded for the following reasons aligned with our a-priori criteria: no pre-post or comparative measurement of attitudes (*n* = 16); single-discipline cohorts only (*n* = 9); population not eligible (postgraduate/licensed professionals only) (*n* = 8); outcomes not eligible (no quantitative attitudinal change reported) (*n* = 8); conducted outside LMIC settings (*n* = 7); and conference abstract/insufficient data for extraction (*n* = 5).

Five studies met all inclusion criteria and were included in the synthesis. These were conducted in Brazil, Iran, India, Lebanon, and Pakistan.

**Fig. 1:**
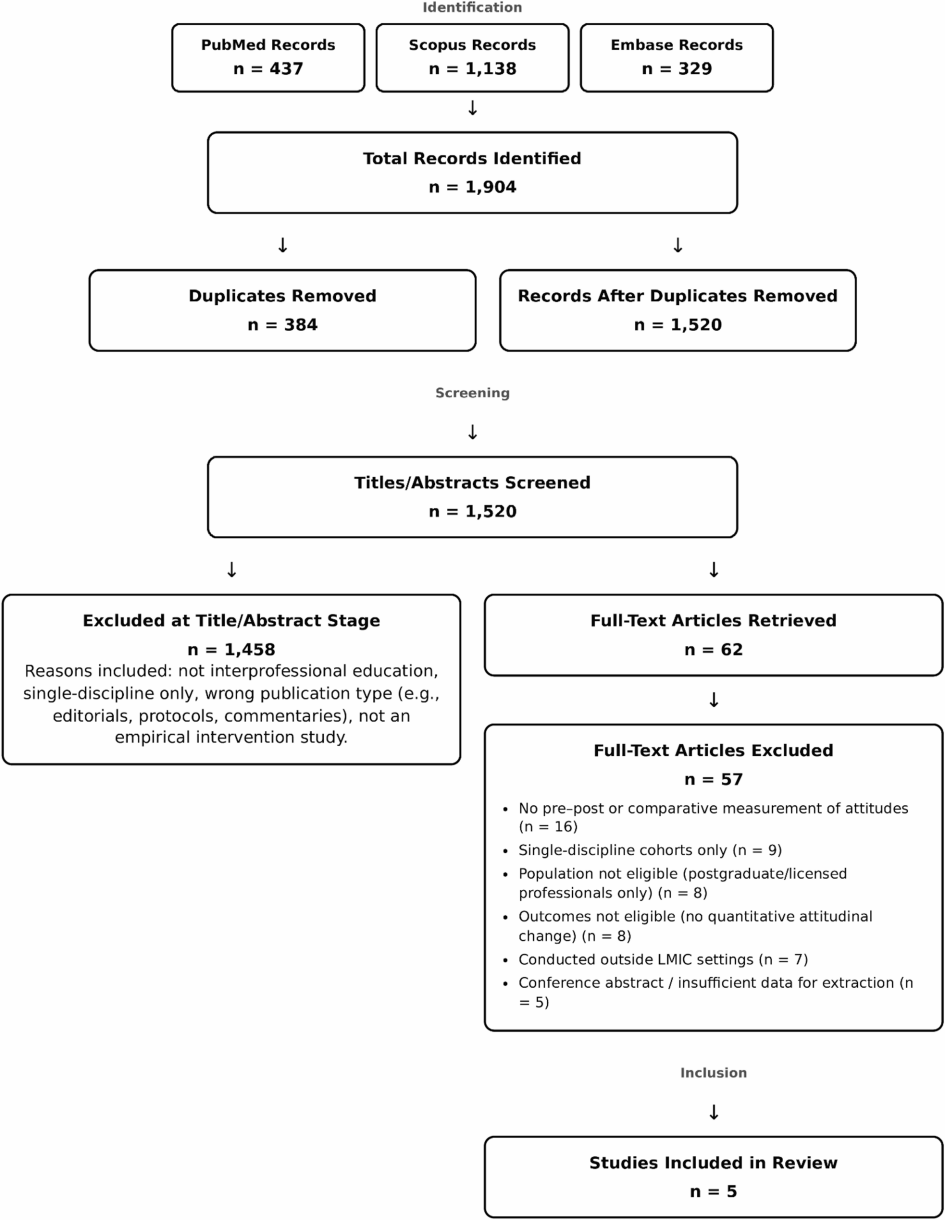
PRISMA flow diagram

PRISMA flow diagram encompassing every stage of screening is presented as Fig. 1.

Table 1 presents the characteristics of the five included studies, conducted between 2017 and 2025 in Brazil, Lebanon, India, Iran, and Pakistan, enrolling a total baseline sample of 396 participants from multiple health professions, most commonly medicine and nursing. All studies used a pre-post design without a concurrent control group. Intervention formats ranged from single-day workshops to multi-week clinical placements and a year-long postgraduate course, delivered in classroom, blended, or ward-based settings. Group sizes varied from pairs to small groups of up to nine students. Attitudes were measured using a mix of validated tools (IEPS, IPAS, RIPLS) and custom questionnaires, with four studies reporting significant positive change in overall attitudes, particularly in domains such as teamwork, role understanding, and communication; and one reporting mixed results with decline in a specific domain.

**Table 1:** Characteristics of included studies [19–23]

Author (Year)	Country/Income Level (WB)	Participants & Disciplines	Sample Size (*N*)	Study Design	Intervention Description	Duration/Frequency	Outcome Measure(s)	Follow-up Period	Main Attitudinal Outcome
Blanco-Vieira et al. (2017) [19]	Brazil/UMIC	Medical, Nursing	111	Pre-post without control	Case-based workshops on collaborative practice	3 sessions	IEPS	Immediate post-test	↑ IEPS subscales (*p* < 0.05)
Dabaghzadeh et al. (2017) [20]	Iran/UMIC	Pharmacy, Nursing	39	Pre-post without control	Ward-based collaborative training rounds	4 weeks	RIPLS	Immediate post-test	↑ RIPLS teamwork subscale
Ray et al. (2021) [21]	India/LMIC	MBBS, BSc Nursing, BPT	90	Pre-post without control	Simulation-based IPE on patient care scenarios	1 day	RIPLS	Immediate post-test	↑ Total RIPLS score (*p* < 0.05)
Sakr et al. (2022) [22]	Lebanon/LMIC	Medical, Nursing, Physiotherapy	72	Pre-post without control	Problem-based IPE on clinical decision-making	2 weeks	RIPLS	Immediate post-test	↑ Total RIPLS score; role understanding improved
Bruce et al. (2025) [23]	Pakistan/LMIC	Medical, Nursing, Pharmacy	84	Pre-post without control	Community health clinic-based IPE rotation	2 weeks	ATHCTS	Immediate post-test	↑ Positive attitudes towards teamwork

*WB *World Bank, *UMIC *Upper-middle-income country, *ATHCTS *Attitudes Toward Health Care Teams Scale

### Population and context

Table 2 outlines the demographic and contextual characteristics of participants across the five included studies. Sample sizes analyzed ranged from 39 in Brazil’s postgraduate mental-health specialization to 111 in Lebanon’s semester-long IPE course, with attrition varying from 0% in the Indian and Pakistani studies to 57% in Lebanon. Female representation was high where reported, ranging from 58% in India to 85% in Brazil. Academic levels varied, with most participants being undergraduates, except in Brazil, where all were multi-professional postgraduates. Recruitment methods included voluntary participation in three studies and course-mandated enrolment in Lebanon; only Pakistan reported explicit year-wise distribution. Instruction was delivered in English in most settings, except Brazil and Iran, where Portuguese and Persian/English mixes were used. Notable contexts included rural or tribal delivery in India, a busy tertiary-care hospital in Pakistan, and the first documented large-scale IPE integration in Middle Eastern medical curricula in Lebanon.

**Table 2:** Population demographics & study context [16, 19–23]

Study ID	Country/WB Income Level	Institution Type/Location	Eligible *N*	Recruited *N* (Baseline)	Analyzed *N*	Attrition %	Recruitment Method	Key Demographics	Program Mix	Prior IPE	Language	Motivation/Ethics/Incentive	Notable Context/Notes
Blanco-Vieira et al., 2017 [19]	Brazil/Upper middle	Federal public university, urban (São Paulo)	54	54	39	28	NR	85% female; mean age 34.8 y; multi-professional postgraduates (psychiatrists 39%)	11 disciplines; psychiatry dominant	Majority had prior clinical contact; formal IPE NR	Portuguese	Ethics approved; written consent; no incentives	360-h interprofessional mental-health specialization
Dabaghzadeh et al., 2017 [20]	Iran/Upper middle	Public university-affiliated teaching hospital, urban (Kerman)	NR	NR	NR	NR	Voluntary (prospective cohort)	Gender/age NR; final-year medical and pharmacy students	Medicine & Pharmacy (ratio NR)	None	Persian/English mix	Ethics approved; written consent; no incentives	Inpatient infectious-disease ward
Ray et al., 2021 [21]	India/Lower middle	Private teaching hospital campus, rural/tribal (Wayanad, Kerala)	NR	96	96	0	Not described	58% female; male/female by profession given; age NR	Balanced 1:1:1 (Medicine, Nursing, Physiotherapy)	None stated	English	Voluntary; ethics approved; written consent; no incentives	Postnatal care IPE in hierarchical Indian setting
Sakr et al., 2022 [22]	Lebanon/Upper middle	Research-intensive private university, urban (Beirut)	346	260	111	57	Course-mandated enrolment	Gender/age NR; medicine, nursing, MPH	Medicine 54%, Nursing 25%, Public Health 22%	Prior IPE NR; high baseline scores	English	Mandatory curricular course: ethics approved; verbal consent; no incentives	First large IPE in Middle East medical curricula
Bruce et al., 2025 [23]	Pakistan/Lower middle	Public tertiary-care teaching hospital, urban (Lahore)	NR	86 (43 nursing, 43 medicine)	86	0	Convenience sampling	76.7% female; age 18–26 y; year 2 (31%), year 3 (36%), final year (33%)	Balanced 1:1 (Medicine, Nursing)	None	English	Voluntary; ethics approved; written consent; no incentives	Course embedded in busy teaching hospital

*MPH *Master of Public Health. “Eligible N” refers to the number of students meeting inclusion criteria prior to recruitment; “Analyzed N” reflects participants included in final analysis. Attrition % calculated as (Recruited N - Analyzed N) ÷ Recruited N × 100

Table 3 summarizes the design and delivery of the IPE interventions across the five included studies. Intervention duration ranged from single-day workshops (India, Iran) to multi-week or semester-long programmes (Pakistan, Lebanon) and a 360-hour postgraduate course (Brazil). Formats included classroom-based didactic sessions, case-based learning, simulation, ward-based clinical placements, and blended approaches. Group sizes within activities varied from small interprofessional teams of 6–8 students to larger mixed-discipline cohorts. Facilitators were drawn from multiple professions in all studies, with varying degrees of structured faculty training in IPE. All interventions targeted interprofessional competencies such as teamwork, communication, and understanding of roles, though emphasis differed by study.

**Table 3:** IPE intervention characteristics [19–23]

Study ID	Intervention Name†	Type	Framework/Model	Duration	Intensity/Schedule	Delivery Format	Setting	Group Size	Professions Included	Key Learning Methods	Assessment Methods	Facilitator Type	Facilitator Training	Clinical Focus	Patient/Case Type
Blanco-Vieira et al., 2017 19]	Child & Adolescent Mental Health Specialization Course (CESMIA)	Post-graduate course	Problem-based learning model	360 h total	Schedule NR (delivered across academic year)	Face-to-face	University classrooms & small-group rooms	Small groups (size NR) from cohort of 39	Lectures, small-group PBL case discussions	Summative - KAP survey pre/post	Course tutors/faculty	NR	Child & adolescent mental health	Paper/discussion cases	--
Dabaghzadeh et al., 2017 [20]	Infectious-disease ward IPE placement	Clinical placement	NR	2 weeks	Daily 4 h (08:00–12:00)	Face-to-face clinical	In-patient infectious-disease ward	Pairs of 2 (1 Pharm + 1 Med)	Bedside care, medication review, daily rounds	Summative - RIPLS pre/post	ID specialist ་ clinical pharmacist	NR	Infectious diseases	Real in-patients	--
Ray et al., 2021 [21]	IPE learning experience in Post-natal Care	Mixed (didactic + case-based + workplace)	NR	NR (multi-session module)	2 didactic talks, 3 case-sessions, ward-based teamwork (period NR)	Blended (classroom ་ ward)	Classroom & postnatal ward	6 students/team; 8 teams/batch	Medicine, Nursing, Physiotherapy	Lectures, case vignettes, small-group planning, bedside care, poster presentation	Retro-prequestionnaire ་ formative poster	Faculty (obstetrics, nursing, physio)	NR	Postnatal maternal care	Real ward patients & paper vignettes
Sakr et al., 2022 [22]	IPE & Collaboration (IPEC) course	Case-based course	IPEC competency framework	≈ 3 months (one semester)	Small-group discussion once/week (exact hours NR)	Face-to-face	Classroom small-group rooms	9 students (4–5 Med, 2–3 Nurs, 2 MPH)	Case-based collaborative learning	Summative - IPAS pre/post	Faculty from three schools	Yes - multiple preparatory workshops	Public-health topics (smoking cessation, HIV, cardiac care, etc.)	Hypothetical paper cases	--
Bruce et al., 2025 [23]	“Structured IPE module” (title NR)	Mixed (case-based + simulation)	NR	4 weeks	Weekly sessions, hours NR	Face-to-face	Classroom sessions ་ simulation lab	6–8 students	Medicine & Nursing under-graduates (1:1)	Scenario-based case discussions, team simulations, guided reflection	Summative - IEPS pre/post survey	Faculty (medicine & nursing)	NR	General teamwork & patient-centered care	Simulated scenarios

Missing items are coded “NR”; no study stated an explicit social-psychological theory (e.g., Contact or Social-Identity) beyond the IPEC competency framework in Sakr et al., 2022. The Lebanese IPEC course is the only programme that explicitly documented structured faculty workshops before delivery, fulfilling best-practice recommendations for facilitator preparation

### Risk of bias

**Fig. 2:**
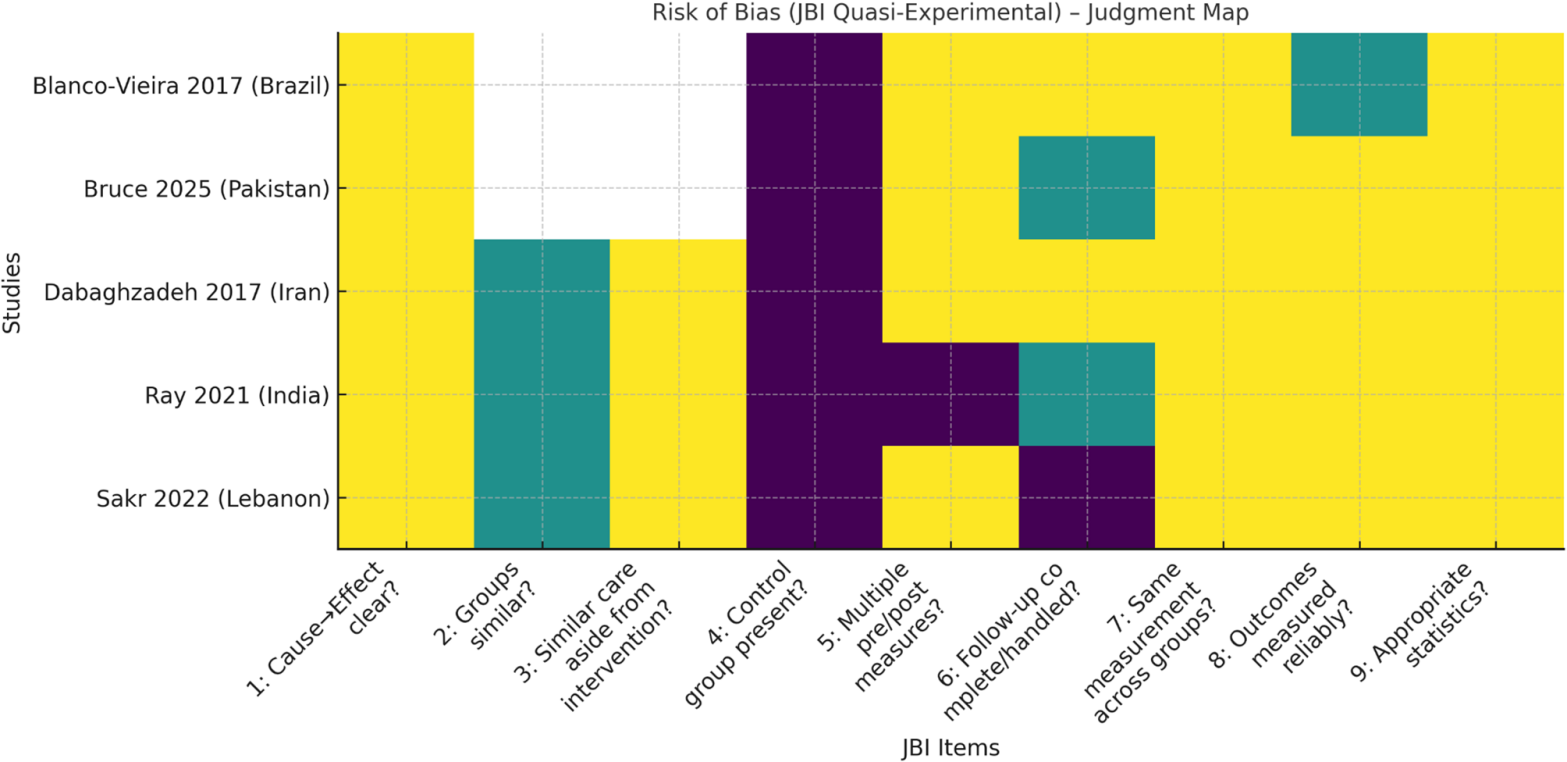
Risk of Bias (JBI) map [16, 19–23]. Colour-map of JBI Critical Appraisal Checklist ratings for the five included studies, where green = “Yes/low risk”, yellow =“Unclear/No-unclear”, and red/purple = “No/high risk”; White denotes items not applicable

All five included studies were appraised using the JBI Critical Appraisal Checklist for Quasi-Experimental Studies, as each employed a non-randomized, pre-post design without a concurrent control group [16]. Table 4 presents the item-by-item judgments alongside brief justifications and an overall verdict for each study, while Fig. 2 displays the same data as a colour-coded map. Across studies, risk was most often introduced by the absence of a control arm (JBI Item 4), unclear reporting on baseline group comparability (Item 2) and incomplete follow-up or limited handling of attrition (Item 6). Measurement reliability was generally acceptable, with three studies using validated attitude scales (IEPS, IPAS, RIPLS) and two relying on tools with limited or no prior validation [10–12]. Appropriate statistical analyses for within-group comparisons were reported in all cases. Based on these criteria, three studies were judged at *moderate* risk of bias, while two (Ray 2021; Sakr 2022) were judged at *serious* risk, mainly due to retrospective-predesign or substantial attrition [21, 22]. 

**Table 4:** Risk of bias summary table with justification and verdict [16, 19–23]

Study ID	JBI1: Cause→Effect clear?	JBI2: Groups similar?	JBI3: Similar care aside from intervention?	JBI4: Control group present?	JBI5: Multiple pre/post measures?	JBI6: Follow‑up complete/handled?	JBI7: Same measurement across groups?	JBI8: Outcomes measured reliably?	JBI9: Appropriate statistics?	Justification (summary)	Final verdict
Blanco-Vieira et al. (2017) [19]	Yes	N/A	N/A	No	Yes	Yes	Yes	No/Unclear	Yes	Uncontrolled pre/post; partial follow-up; non-validated KAP tool elevates measurement bias; stats appropriate.	Moderate risk
Dabaghzadeh et al. (2017) [20]	Yes	Unclear	Yes	No	Yes	Yes	Yes	Yes	Yes	Prospective cohort; no control; validated RIPLS; high completion; appropriate non-parametric analysis.	Moderate risk
Ray et al. (2021) [21]	Yes	Unclear	Yes	No	No	Unclear	Yes	Yes	Yes	Retro‑pre (no true baseline); no control; handling of missing data unclear; stats align with within-subject design.	Serious risk
Sakr et al. (2022) [22]	Yes	Unclear	Yes	No	Yes	No	Yes	Yes	Yes	Pre/post with substantial attrition; no control; validated IPAS; appropriate paired analysis.	Serious risk
Bruce et al. (2025) [23]	Yes	N/A	N/A	No	Yes	Unclear	Yes	Yes	Yes	Uncontrolled pre/post; attrition handling unclear; validated IEPS with appropriate paired tests.	Moderate risk

### Quantitative synthesis (SWiM vote-count approach)[18]

Table 5 details the attitudinal outcome measures used and their key properties. Three studies employed validated instruments-the IEPS, IPAS, or RIPLS-while two used custom or adapted questionnaires with partial or no validation evidence [10–12]. All tools generated numerical pre- and post-intervention scores, allowing calculation of change over time. The number of items per scale ranged from 9 to 35, with response formats typically on 5- or 6-point Likert scales [24]. Domains covered included teamwork, communication, perceptions of professional roles, and readiness for interprofessional learning. Timing of post-intervention measurement was immediate in all studies.

**Table 5:** Outcome measurements and instruments [19–23]

Study ID	Primary instrument (full name)	Code	Total items	Sub-scales/domains assessed	Response scale & score range	Original validation context	Local validation reported?	Language of administration	Reliability (α) reported	Measurement schedule	Secondary outcomes
Blanco-Vieira et al., 2017] [19]	Knowledge-Attitude-Practice questionnaire (self-developed)	KAP	NR (18 attitude items within larger tool)	Knowledge; Attitudes; Practice domains (item-level details NR)	NR (Likert type stated, anchors NR)	Not previously validated (new tool)	No	Portuguese (assumed; not specified)	NR	Pre-course and post-course (end of 360-h programme)	Knowledge & Practice sub-scores
Dabaghzadeh et al., 2017 [20]	Readiness for Interprofessional Learning Scale	RIPLS	19	Teamwork & Collaboration; Professional Identity (positive & negative); Roles & Responsibilities	5-pt Likert (1 = Strongly Disagree → 5 = Strongly Agree); score range NR	Widely used with under- & post-graduate health students internationally	Persian version previously validated; current study did not re-validate (No)	Persian	Cronbach’s α NR (instrument validity noted)	Pre-workshop and immediate post-workshop (same day)	None
Ray et al., 2021 [21]	Custom retro-pre-IPE attitude questionnaire	Retro-Pre	35	Comfort questioning peers; Reliability & Trust (plus other attitude facets - item list NR)	NR (Likert type implied but not specified)	Developed with University of Houston IPE faculty	Unclear	English (online survey)	NR	Single administration post-course capturing retrospective “pre” & “post” ratings (retrospective-predesign)	Open-ended qualitative feedback
Sakr et al., 2022 [22]	Interprofessional Attitudes Scale	IPAS	27	Team Roles & Responsibilities; Patient–Centeredness; Interprofessional Biases; Diversity & Ethics; Community-Centeredness	7-pt Likert (1 = Strongly Disagree → 7 = Strongly Agree); one item reverse-scored; total-score range NR	Norris et al., 2015, USA students (cited)	No	English (instruction language)	NR (tool described as “validated”)	Baseline first course session & 3 months later at course completion	None
Bruce et al., 2025 [23]	IPE Perception Scale	IEPS	NR	Competency & Autonomy; Perceived Need for Co-operation; Perception of Actual Co-operation	5-pt Likert (1 = Strongly Disagree → 5 = Strongly Agree); total-score range NR	Developed for USA health-professions students (cited in text)	No	English (paper, supervised)	“Strong internal reliability” - exact α NR	Pre-module (week 0) & immediate post-module (week 4)	None

**Fig. 3:**
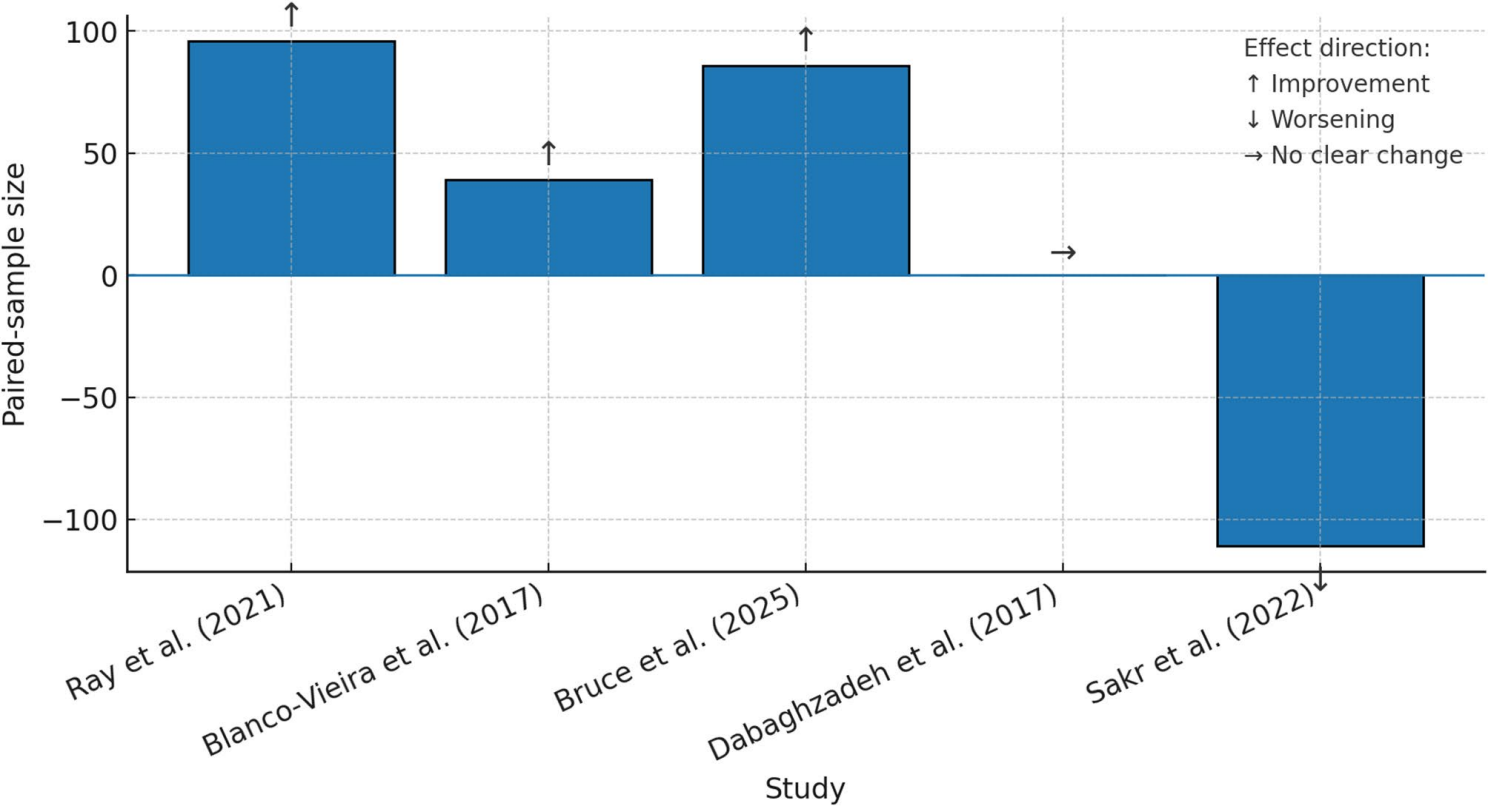
- Harvest plot illustrating effect direction (↑ improvement, ↓ worsening, → no clear change) and paired-sample size for each study

We coded the direction of change (↑ improvement, ↓ worsening, ↔ no change) for each primary attitude outcome, following SWiM guidance and presented findings in a harvest plot. Effect-direction coding revealed that four of the five included LMIC studies reported statistically significant positive shifts in IPE attitudes (↑, *p* ≤ 0.001). These studies were Bruce 2025 (IEPS), Ray 2021 (retro-pre survey), the medical subscales of Dabaghzadeh 2017 (RIPLS), and Blanco-Vieira 2017 (KAP). In contrast, Sakr 2022 showed a small but significant decline in the IPAS patient-centeredness domain (↓, *p* = 0.003), an effect that coincided with the highest attrition rate (68%), suggesting potential response bias. It is noteworthy that while the medical subscales in Dabaghzadeh 2017 were positive, the overall attitude score for the study remained neutral (↔). This study also highlighted context-sensitive outcomes, as clinical placements in Iran produced profession-specific effects: a positive shift for medical students (↑) versus no change for pharmacy students (↔). (Fig. 3)

### Qualitative synthesis

Table 6 summarizes qualitative data from open-ended survey questions or focus group discussions conducted in four of the five included studies. The most frequently reported themes were improved teamwork, better understanding of other professional roles, and enhanced communication. Other themes included increased confidence in collaborative settings, greater appreciation of shared decision-making, and recognition of mutual respect among professions. In two studies, participants highlighted the practical relevance of IPE to clinical practice. A minority of comments reflected logistical challenges or perceptions of unequal participation among disciplines.

**Table 6:** Qualitative feedback & thematic findings [19–23]

Study ID	Qualitative methods used	Qualitative sample (*n*)	Positive feedback themes	Challenges/barriers reported	Learning outcomes described	Behavior/professional-identity impact	Implementation acceptability & suggestions	Faculty observations
Blanco-Vieira et al., 2017 [19]	None (KAP survey; narrative reflection in discussion)	39 completed both KAP surveys	Participants valued small-group case discussion supervised by child-mental-health tutors to “build a joint solution”	No validated tool; small sample; absence of follow-up; convenience sampling	Reported 17% attitude gain, 14% practice gain reinforcing relevance of inter-professional courses	Older, more experienced professionals gained most knowledge (identity reinforcement)	Participants felt course length (360 h) and multiple interactive methods were important for change	NR
Dabaghzadeh et al., 2017 [20]	None (prospective cohort with RIPLS; qualitative remarks in discussion)	NR	Students reported increased knowledge and eagerness for more IPE courses	Extracurricular timing, busy clinical schedules, time-consuming two-week rotation	Improved teamwork & communication (author interpretation)	NR	Recommend embedding IPE in curricula and providing suitable environment	NR
Ray et al., 2021 [21]	Open-ended questions analyzed thematically	96 students; 96 responses, 40-31−25 citing top themes	Teamwork (42%), Communication (32%) & Knowledge of other roles (26%) with illustrative quotes such as “The greatest thing was working together … in a clinical scenario”	None noted by students; authors discuss cultural hierarchies as potential barrier	Enhanced respect, clarity on roles, confidence in inter-disciplinary dialogue	Students reported “now I fully comprehend what their roles are” indicating shift in professional identity	Small-group discussion and real ward work were rated “most important” parts of experience	Facilitators observed that questioning across professions-built trust
Sakr et al., 2022 [22]	None (pre/post IPAS survey only)	NR	NR (study focused on attitude scores)	Resource-intensive course; 40 faculty/year, curricular change across three faculties, heterogeneity of student experience	NR	NR	Authors highlight need for protected time and faculty development to sustain course	NR
Bruce et al., 2025 [23]	None reported (quantitative survey only; no open-ended items)	NR	Students’ discussion section notes “significant gains in perceived cooperation, shared decision-making and understanding of roles”	Single-site setting, no long-term follow-up, limited generalizability	Better appreciation of teamwork and mutual respect	Final-year students showed highest post-scores, suggesting stronger professional identity integration	NR (course judged effective but no student suggestions captured)	NR

### Mixed-methods integration and heterogeneity

Aligning quantitative vote-count data with qualitative themes suggests that interventions emphasizing small-group dialogue and real clinical exposure (Pakistan, India, Iran) were more likely to produce positive attitude shifts, whereas resource-intensive, semester-long courses with high attrition (Lebanon) risk dilution of impact. Professional hierarchy and lack of protected time emerged as plausible explanations for heterogeneity.

### Evidence gap map

**Fig. 4:**
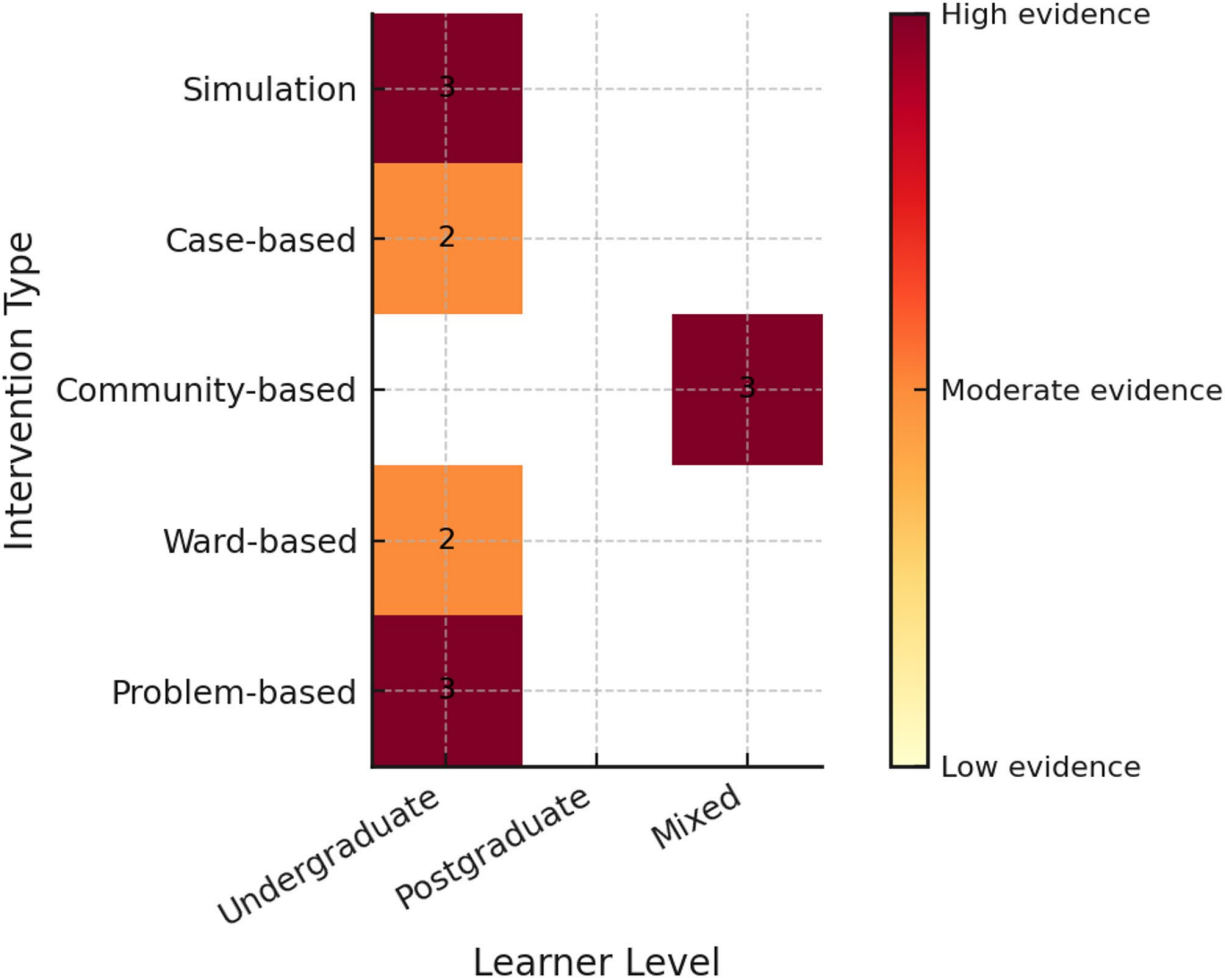
Evidence gap map of IPE interventions by learner level. Numeric values within cells indicate the strength of available evidence (1 = low, 2 = moderate, 3 = high). Empty cells represent areas with no identified studies. Notably, no studies evaluated simulation-based IPE at postgraduate or mixed learner levels, and no included study assessed outcomes beyond six months post-intervention

Plotting of Intervention type against learner level was as seen in Fig. 4. Empty cells underscore missing evidence for simulation-based IPE and long-term (> 6 month) follow-up in LMIC contexts.

### Certainty of evidence

Using the GRADE adaptations for narrative synthesis, overall certainty was low due to risk of bias (uncontrolled designs), imprecision (small samples) and inconsistency (one study showing decline) [17]. Nevertheless, direction-of-effect consistency in four studies allowed moderate confidence that brief, well-facilitated IPE can improve professional attitudes in LMIC undergraduate settings. **(**Table 7**)**

**Table 7:** GRADE summary of Findings - Attitudes toward IPEC after IPE among LMIC Health‑Professions students [17]

Outcome (construct)	No. of studies (design)	Population & setting	Comparator	Findings (direction & consistency)	Certainty of evidence (GRADE)	Reasons for rating (domain‑wise justification)
Overall attitude toward interprofessional collaboration after IPE (primary)	5 studies (all non‑randomized pre-post; 3 with validated tools: IEPS/IPAS/RIPLS; 2 with bespoke/KAP tools)	Undergraduate and postgraduate learners in Pakistan, Lebanon, India, Iran, Brazil; classrooms, blended, and clinical placements	Within‑group pre vs. post	4 of 5 studies showed significant improvement (↑); 1 study reported a small decline limited to an IPAS subdomain (patient‑centredness). Direction of effect coherent across contexts; largest attrition occurred in the study with the decline.	Low	Risk of bias - serious (downgrade 1 level): all are uncontrolled pre-post; 1 retrospective‑pre; one with substantial attrition. Inconsistency - not serious: 4↑ vs. 1↓ (limited to a single subdomain) with a plausible explanation (attrition/response bias). Indirectness - not serious: LMIC students; IPE interventions match review question; three validated attitude scales used. Imprecision - not serious: total analyzed *N* ≈ 383; although effect sizes/SDs often missing, direction is consistent and sample adequate for vote‑count SWiM. Publication bias - suspected but not rated: small studies and positive direction could suggest bias, but evidence insufficient to downgrade further.
Patient‑centredness subdomain of attitudes (sensitivity outcome)	1 study (IPAS domain analysis)	Undergraduate students in a semester‑long IPE course (Lebanon)	Within‑group pre vs. post	Small but statistically significant decline (↓) confined to patient‑centredness; other IPAS domains not consistently decreased.	Very low	Risk of bias - serious: high attrition and no control; domain‑specific post‑hoc focus. Inconsistency - very serious: effect seen in a single study/domain without replication. Indirectness - not serious. Imprecision - serious: single study with attrition and no precision estimates for domain effect. Publication bias - suspected. (Multiple downgrades yield very low.)

Starting level set per GRADE for non‑randomized pre-post designs (observational), i.e., Low; downgrades and additional downgrades applied by domain using narrative‑synthesis guidance. No upgrading was applied because large, precise, and consistent magnitude effects or dose‑response patterns were not demonstrated across studies

## Discussion

IPE in LMICs remains inconsistently implemented despite long-standing global calls for collaborative practice [1–3]. This review synthesized evidence from five quasi-experimental studies in India, Pakistan, Brazil, Iran, and Lebanon, all reporting positive or neutral shifts in student attitudes toward collaboration [19–23]. These findings support the WHO’s Framework for Action on IPE & Collaborative Practice and align with the IPEC’s core competency updates, which emphasize values/ethics, roles and responsibilities, interprofessional communication, and teamwork [1–3]. 

The greatest attitudinal gains occurred when IPE was embedded in authentic clinical contexts and included diverse professional groupings. Ray et al. (2021) in rural India and Bruce et al. (2025) in Pakistan demonstrated significant improvements across all measured domains when interventions centered on ward-based care or realistic case simulations [21, 23]. Blanco-Vieira et al. (2017) reported the largest relative improvement (+ 17% in attitudes) following a prolonged, 360-hour course for mental-health professionals, suggesting that both intervention intensity and sustained interaction enhance outcomes [19]. Conversely, Sakr et al. (2022) found only patient-centredness improved, likely due to high baseline scores and a ceiling effect commonly reported in IPE evaluations [7, 22]. 

Risk-of-bias was generally moderate to serious: all studies used uncontrolled pre/post designs, relied on self-report instruments (e.g., RIPLS, IEPS, IPAS), and two had notable attrition (e.g., 57% unmatched pairs in Sakr et al., 2022) [10–12, 16, 22]. Certainty of evidence was downgraded for risk of bias, imprecision, and inconsistency. Nevertheless, qualitative data from India and Brazil reinforced quantitative gains, with participants citing improved trust, role clarity, and communication-consistent with recent systematic reviews identifying these as core intermediate outcomes of IPE [5, 9]. These findings can inform curriculum design in LMICs by advocating for low-cost, clinically embedded IPE programs that leverage existing resources to foster collaborative attitudes early in training, with policy implications for national health education bodies to prioritize IPE integration for improved team-based care.

Profession-specific responsiveness emerged as a key nuance. Dabaghzadeh et al. (2017) found significant improvement among medical but not pharmacy students after a shared inpatient rotation [20]. Possible explanations include entrenched role perceptions, uneven clinical responsibilities, or smaller pharmacy sample sizes. Literature reports similar profession-specific variations, which underscore the need for tailored role-clarification and feedback strategies [3, 7]. 

Implementation lessons for LMICs are clear. Curriculum-aligned, low-cost approaches-such as structured case discussions, ward-based team care, and small-group simulations-proved feasible and acceptable in hierarchical, resource-constrained environments [21, 23]. These approaches reflect WHO’s recommendation for context-fit, scalable IPE and align with IPEC’s 2023 emphasis on adaptable core competencies [1, 3]. 

Looking forward, LMIC-IPE research should progress beyond immediate post-intervention attitudes to assess sustained behavioral change and patient-level outcomes. Cochrane reviews and recent meta-analyses demonstrate such downstream effects are possible, but robust controlled designs, harmonized outcome measures, and longitudinal follow-up are essential to determine both effectiveness and cost-effectiveness in resource-constrained health systems [7, 8]. 

### Limitations

Evidence being generated from LMIC in IPE is not much. Besides the limited number of studies making it to the final review, synthesis is constrained by the methodological quality of the included studies as well. All used uncontrolled pre/post designs, limiting causal inference and leaving findings vulnerable to confounding from concurrent curricular experiences. Reliance on self-reported attitudinal instruments introduces social desirability bias, particularly in mandatory or graded courses.

Sample sizes were modest (39–111 participants per study), and attrition was substantial in some cases (e.g., 57% unmatched pairs in one study), reducing precision and increasing risk of attrition bias. The heterogeneity of participant profiles, intervention intensity, and measurement tools precluded meta-analysis and limited comparability. Differences in cultural norms, health-system structures, and academic calendars across the five LMIC contexts further constrain generalizability.

No study included long-term follow-up, leaving the sustainability of observed attitudinal gains unknown. Finally, publication bias cannot be ruled out, as small-scale LMIC-IPE projects with null results are less likely to be reported. Addressing these limitations will require multicenter, controlled trials using harmonized tools, robust handling of missing data, and mixed-methods approaches to capture context-specific facilitators and barriers.

### Recommendations and future directions

This review demonstrates that IPE in LMIC contexts can positively influence learners’ attitudes toward collaboration. The most significant gains were observed in interventions that were contextually relevant, clinically embedded, and inclusive of multiple professional groups, particularly when activities mirrored real-world patient care. These findings suggest that IPE has the potential to foster teamwork and bridge professional silos within resource-constrained, hierarchically structured health systems.

The current body of evidence is limited by several factors, including small sample sizes, methodological heterogeneity, and the frequent absence of control groups. Furthermore, the reliance on self-report measures and short-term follow-up periods reduces the certainty of observed effects, making it difficult to confirm long-term behavioral changes or patient-level benefits.

Lessons from LMIC contexts, such as scalable low-cost models, are applicable to HICs facing resource variability. International collaborations, like the International Interprofessional Collaborative Office Rounds (iiCOR) program connecting professionals across Global North and South via distance technology, could provide further insights for cross-contextual IPE implementation [25]. 

## Supplementary Information

Supplementary Material 1

## Data Availability

All data generated or analyzed during this study are included in this published article and its supplementary information files. Additional datasets used and/or analyzed during the current study are available from the corresponding author on reasonable request.
