# Supplementary material for: Bridging silos on a budget: how interprofessional education shapes collaborative attitudes across low-and middle-income countries - a systematic review
Source: BMC Med Educ. 2025 Nov 14;25:1602. doi: 10.1186/s12909-025-08203-6 (PMC12619399; doi:10.1186/s12909-025-08203-6)
Supplement: Supplementary file 1 — Supplementary Material 1 [file 12909_2025_8203_MOESM1_ESM.docx]

# **Appendix 1: List of abbreviations**

IPE - Interprofessional education

LMICs - Low- and middle-income countries

WHO - World Health Organization

IPEC - Interprofessional Education Collaborative

PRISMA-P - Preferred Reporting Items for Systematic Review and Meta-Analysis Protocols

JBI - Joanna Briggs Institute

IEPS - Interdisciplinary Education Perception Scale

IPAS - Interprofessional Attitudes Scale

RIPLS - Readiness for Interprofessional Learning Scale

KAP - Knowledge-Attitude-Practice

PICO - Population-Intervention-Comparator-Outcome

GRADE - Grading of Recommendations, Assessment, Development and Evaluations

SWiM - Synthesis Without Meta-analysis

UMIC - Upper-middle-income country

NR - Not reported

WB - World Bank

# **Appendix 2: Detailed Search Strategies**

## **PubMed** (last accessed on August 10, 2025)

## (

IPE[tiab] OR "interprofessional education"[Mesh] OR "interprofessional education"[tw]

OR "inter-professional education"[tw] OR "interdisciplinary education"[tw]

OR "inter-disciplinary education"[tw] OR "multiprofessional education"[tw]

OR "multidisciplinary education"[tw] OR "multi-professional education"[tw]

OR "multi-disciplinary education"[tw]

)

AND

(

"Students, Health Occupations"[Mesh] OR student*[tiab] OR trainee*[tiab] OR undergrad*[tiab]

OR postgraduate*[tiab] OR intern*[tiab]

)

AND

(

"Developing Countries"[Mesh] OR "Global Health"[Mesh:noexp]

OR (low-income countr*[tiab] OR lower-middle-income countr*[tiab] OR upper-middle-income countr*[tiab] OR LMIC*[tiab] OR "low- and middle-income"[tiab] OR "resource-limited"[tiab] OR "resource-constrained"[tiab] OR "global south"[tiab])

OR (Afghanistan OR Bangladesh OR Benin OR Bhutan OR Bolivia OR Brazil OR Cambodia OR Cameroon OR China OR Colombia OR Congo OR Côte d'Ivoire OR Egypt OR Ethiopia OR Ghana OR India OR Indonesia OR Iran OR Iraq OR Jordan OR Kenya OR Laos OR Lebanon OR Morocco OR Myanmar OR Nepal OR Nigeria OR Pakistan OR Peru OR Philippines OR Rwanda OR Senegal OR South Africa OR Sri Lanka OR Tanzania OR Thailand OR Tunisia OR Uganda OR Ukraine OR Vietnam OR Zambia OR Zimbabwe)[tiab]

)

AND (2010/01/01:2025/08/10[dp])

AND (english[lang])

## **Scopus** (last accessed on August 10, 2025)

With additional language (English) and year-duration cap (2010 to August 10, 2025):

TITLE-ABS-KEY(

(

(interprofessional OR "inter-professional" OR interdisciplinary OR "inter-disciplinary" OR multidisciplinary OR "multi-disciplinary" OR multiprofessional OR "multi-professional")

W/3 (education OR learning OR training OR curriculum OR simulation OR course OR school)

)

AND

(student* OR trainee* OR undergraduate* OR postgraduate* OR intern*)

AND

(

"developing countr*" OR "low-income countr*" OR "low and middle income" OR lmic* OR "resource-limited" OR "resource-constrained" OR "global south"

OR afghanistan OR bangladesh OR benin OR bhutan OR bolivia OR brazil OR cambodia OR cameroon OR china OR colombia OR congo OR "cote d'ivoire" OR egypt OR ethiopia OR ghana OR india OR indonesia OR iran OR iraq OR jordan OR kenya OR laos OR lebanon OR morocco OR myanmar OR nepal OR nigeria OR pakistan OR peru OR philippines OR rwanda OR senegal OR "south africa" OR "sri lanka" OR tanzania OR thailand OR tunisia OR uganda OR ukraine OR vietnam OR zambia OR zimbabwe

)

)

## **Embase** (last accessed on August 10, 2025)

(

(ipe OR "interprofessional education" OR "inter-professional education"

OR "interdisciplinary education" OR "inter-disciplinary education"

OR "multiprofessional education" OR "multi-professional education"

OR "multidisciplinary education" OR "multi-disciplinary education"

OR (interprofessional NEAR/3 (education OR learning OR training))

):ti,ab,kw

)

AND

(

(student* OR trainee* OR undergrad* OR postgraduate* OR intern*

OR "health profession* student*" OR "health occupations student*"

OR "health science* student*" OR "medical student*" OR "nursing student*"

OR "pharmacy student*" OR "dental student*" OR "physiotherap* student*"

):ti,ab,kw

)

AND

(

("developing countr*" OR "global health" OR "low-income countr*"

OR "lower-middle-income countr*" OR "upper-middle-income countr*"

OR LMIC* OR "low and middle income" OR "resource-limited"

OR "resource-constrained" OR "global south"

OR Afghanistan OR Bangladesh OR Benin OR Bhutan OR Bolivia OR Brazil

OR Cambodia OR Cameroon OR China OR Colombia OR Congo

OR "Cote d Ivoire" OR Egypt OR Ethiopia OR Ghana OR India OR Indonesia

OR Iran OR Iraq OR Jordan OR Kenya OR Laos OR Lebanon OR Morocco

OR Myanmar OR Nepal OR Nigeria OR Pakistan OR Peru OR Philippines

OR Rwanda OR Senegal OR "South Africa" OR "Sri Lanka" OR Tanzania

OR Thailand OR Tunisia OR Uganda OR Ukraine OR Vietnam OR Zambia OR Zimbabwe

):ti,ab,kw

)

AND [2010-2025]/py

AND [english]/lim

**Appendix 3: PRISMA Checklist**

| **Section/Topic** | **Item #** | **Checklist item** | **Location where item is reported** |
| --- | --- | --- | --- |
| TITLE | 1 | Identify the report as a systematic review. | Title page |
| ABSTRACT | 2 | See PRISMA 2020 for Abstracts checklist. | Structured Abstract |
| INTRODUCTION | 3 | Describe the rationale for the review in the context of what is already known. | Introduction, para 1–3 |
|  | 4 | Provide an explicit statement of the objective(s) or question(s) the review addresses. | Introduction, end; Methodology—Review question |
| METHODS | 5 | Specify inclusion and exclusion criteria and how studies were grouped for synthesis. | Eligibility Criteria |
|  | 6 | Specify all information sources (databases with dates of coverage, contact with study authors to identify additional studies) and date last searched. | Information Sources and Search Strategy; Grey Literature |
|  | 7 | Present full search strategies for all databases, including any filters and limits used. | Appendix 1: Detailed Search Strategies |
|  | 8 | Specify the methods used to decide whether a study met the inclusion criteria of the review. | Study Selection process |
|  | 9 | Specify the methods used to collect data from reports. | Data Extraction |
|  | 10a | List and define all outcomes for which data were sought. | Data Items |
|  | 10b | List and define all other variables for which data were sought (e.g., participant and intervention characteristics, funding sources). | Data Items |
|  | 11 | Describe any methods used to assess risk of bias in included studies. | Risk-of-Bias Assessment |
|  | 12 | Specify methods used for assessing certainty (or confidence) in the body of evidence. | Certainty Assessment |
|  | 13a | Describe methods of handling data and combining results of studies, if done. | Effect Measures and Data Synthesis |
|  | 13b | Describe any methods used to explore possible causes of heterogeneity. | Effect Measures and Data Synthesis; Qualitative Integration |
|  | 13c | Describe any sensitivity analyses conducted. | Not applicable (no sensitivity analyses conducted) |
| RESULTS | 14 | Give numbers of studies screened, assessed for eligibility, and included, with reasons for exclusions at each stage. | Results—Study selection; Figure 1 PRISMA flow diagram |
|  | 15 | Cite studies that might appear to meet the inclusion criteria, but which were excluded, and explain why they were excluded. | Results—Study selection |
|  | 16a | Present characteristics for which data were extracted for each study. | Tables 1–3 |
|  | 16b | Cite each included study and present its data. | Tables 1–3 |
|  | 17 | Present data on risk of bias for each study. | Table 4; Figure 2 Risk of Bias map |
|  | 18 | Present results of individual studies for all outcomes. | Tables 1–5; Results text |
|  | 19 | Present results of syntheses (summary of findings). | Results—Quantitative synthesis, Qualitative synthesis; Table 7 GRADE Summary |
|  | 20 | Present results of any sensitivity analyses. | Not applicable |
|  | 21 | Present assessments of certainty (or confidence) in the body of evidence. | Table 7; Certainty of evidence section |
| DISCUSSION | 22a | Provide a general interpretation of the results in the context of other evidence. | Discussion, para 1–3 |
|  | 22b | Discuss any limitations of the evidence included in the review. | Limitations section |
|  | 22c | Discuss any limitations of the review processes used. | Limitations section |
|  | 22d | Discuss implications for practice, policy, and future research. | Future Directions; Recommendations |
| OTHER INFORMATION | 23a | Register the review and provide registration information. | Design and Registration |
|  | 23b | Indicate where the review protocol can be accessed. | Design and Registration (OSF DOI link) |
|  | 23c | Describe and explain any amendments to information provided at registration or in the protocol. | Not applicable (no amendments reported) |
|  | 24a | Describe sources of financial or non-financial support for the review. | Funding |
|  | 24b | Describe the role of the funders in the review. | Funding (None declared) |
|  | 25 | Declare any competing interests of review authors. | Conflicts of Interest |
|  | 26 | Provide information about the availability of data, code, and other materials used in the review. | Declaration statement |
